# Comparing the experience of enhanced recovery programme for gynaecological patients undergoing laparoscopic versus open gynaecological surgery: a prospective study

**DOI:** 10.1186/s13741-018-0096-5

**Published:** 2018-06-27

**Authors:** Joanne Lee, Viren Asher, Arun Nair, Victoria White, Catherine Brocklehurst, Martyn Traves, Anish Bali

**Affiliations:** Royal Derby Hospital, Uttoxeter Road, Derby, DE22 3NE UK

**Keywords:** Enhanced recovery programme, Gynaecology, Patients’ experience

## Abstract

**Background:**

Enhanced recovery has been shown to improve patients’ experience after surgery. There are no previous studies comparing patients’ experience between those undergoing laparoscopic and open gynaecological surgery. Therefore, the aim of this prospective study is to compare patients’ functional recovery based on milestones set by the enhanced recovery programme and patients’ satisfaction between the two groups.

**Methods:**

All eligible patients undergoing gynaecological surgery within an enhanced recovery after surgery (ERAS) programme from March to August 2014 were involved in this study. All patients received the questionnaires on admission which were then collected prior to discharge. They were followed up by telephone within 7 days.

**Results:**

Two hundred sixty-three patients were involved. One hundred forty-four questionnaires were returned (54% response rate). Fifty-one percent (*n* = 74) were from the laparoscopic group and 49% (*n* = 70) were from the laparotomy group. In terms of achieving milestones, more patients in the laparotomy group performed the deep breathing exercises (laparoscopic versus open; 66.2% versus 87.1% (*p* = 0.003). The laparoscopic group were more able to eat on day 0, but by day 1, there was no difference between the groups. Both groups were similar in their ability to drink (*p* = 0.98), mobilise (*p* = 0.123) and sit out in a chair (*p* = 0.511). In the laparoscopic group, the patients’ experience was better for pain control (*p* < 0.0001) and nausea control (*p* = 0.003) from recovery to day 1, and they were more able to put on their own clothes (*p* = 0.001) and were more confident in mobilising (*p* < 0.0001) and in going home (*p* < 0.0001). The laparoscopic group had greater patient satisfaction with their pain always being well controlled (*p* < 0.0001) whilst more patients in the laparotomy group reported being satisfied to very satisfied with their overall care on the gynaecology ward (*p* = 0.04). Both groups were equally satisfied with their care from nursing staff (*p* = 0.709) and doctors (*p* = 0.431).

**Conclusion:**

The two groups were in general equally able to achieve the majority of the milestones despite differences in symptoms such as pain, nausea and confidence in mobilising and going home. Pre-operative education can empower patients to engage in their recovery. There is a high level of patient satisfaction in both groups.

## Background

Enhanced recovery after surgery (ERAS) also known as ‘fast track’ surgery is a successful multimodal programme designed to enhance patients’ postoperative recovery without increasing patients’ morbidity (Kehlet and Wilmore 2008). This is achieved with evidence-based care, such as using regional anaesthesia, performing a minimally invasive technique if appropriate, optimising pain control and aggressive postoperative rehabilitation including early oral nutrition and mobilisation. The combination of these approaches reduces the stress response and organ dysfunction and significantly shortens the time to achieve full recovery (Wilmore and Kehlet 2001).

Enhanced recovery is also known to be cost effective by reducing length of stay whilst maintaining improvements in quality of patients’ care (DH, N.I., NCAT, NHS institute 2011). As a result, in 2009, the department of health in England introduced the ‘enhanced recovery programme’ (ERP), and Royal Derby Hospital elected to be one of the pilot hospitals to introduce the scheme. Table 1 shows the elements of enhanced recovery implemented in our unit.

**Table 1:** Elements of enhanced recovery after surgery

Pre-operative	Intra-operative	Post-operative
•*Preadmission counselling •Fluid and carbohydrate loading •No prolonged fasting (clear fluids allowed up to 2 h before surgery) •No/selective bowel preparation (to reduce the risk of post-operative ileus) •Antibiotic prophylaxis •Thromboprophylaxis •**No premedication (by avoiding long acting pre-operative sedative pre-medication, the outcome of ERAS is enhanced by allowing early mobilisation, early oral intake in patients and early catheter removal)	•Short acting anaesthetic agents •Regional analgesia •Avoidance of drains •Avoidance of salt and fluid overload •Patient warming	•Avoidance of NG tubes •Prevention of nausea and vomiting •Minimal opioid analgesia •Use of NSAIDs •Early catheter removal •Early oral nutrition (oral liquids are allowed on the night of surgery and light diet on post-operative day 1 with rapid progression thereafter) •Early mobilisation (day 1 after surgery) •Nurse led follow-up •Audit of compliance and outcome

*At preadmission counselling, patients undergoing a laparotomy had a face to face consultation with a physiotherapist whilst those having a laparoscopic surgery were given a leaflet on exercises that would have been taught by a physiotherapist

** Some patients were given premedication by their anaesthetist

Whilst quality of care within ERAS based on length of stay, complication rates and readmission has been studied, other ways of evaluating effectiveness such as patients’ experience of the programme have not been thoroughly evaluated. Various authors have explored patients’ experience and their satisfaction with ERAS when undergoing colorectal surgery, abdominal hysterectomy and those undergoing surgery for gynaecological cancers (Blazeby et al. 2010; Norlyk and Harder 2009; Norlyk and Harder 2011; Wagner et al. 2004; Wagner et al. 2005; Archer et al. 2014; Philp et al. 2015). They found ERAS to be an effective programme with high patient satisfaction despite early discharge from hospital.

However, there is paucity of data evaluating patients’ functional recovery such as ability to mobilise, eat and drink and dressing oneself in order to return to normal daily function. In addition, there is no data comparing patients’ functional recovery and patients’ satisfaction in laparoscopic and open gynaecological surgery. Therefore, we aim to compare patients’ ability to achieve milestones set by ERAS as parameters of functional recovery and patients’ satisfaction in patients undergoing laparoscopic versus open gynaecological surgery within the ERAS programme.

## Methods

This study was approved by the audit committee at Royal Derby Hospital, and ethical approval was not required. This was a quality assurance project which was evaluated prospectively. Consecutive women undergoing gynaecological surgery in the ERAS programme between 1st of March to 31st of August 2014 were involved in this study. Patients who had a vaginal hysterectomy, pelvic floor repair, urogynaecological surgery and wide local excision of the vulva were excluded from this study. This is because they had different post-operative catheter and drain care that was not congruent with the departmental ERAS protocol. All participants had either a laparoscopic hysterectomy or laparotomy procedure with a transverse or midline incision. Patients were operated by different surgeons who work with the same anaesthetist. The protocol for ERAS was the same for both groups. All patients were cared for on the same ward.

All patients received the questionnaire on the day of admission. Patients were asked to complete the questionnaire daily to capture their true experience on the ward. The main section was divided into three parts. The first part included patients’ impression of pain, nausea, temperature control and their satisfaction of care in recovery. The second part assessed patients’ daily ability to achieve certain milestones set out by the ERAS protocol such as early oral intake, early mobility, achieving independence and the control of pain and nausea. The final section addressed patients’ satisfaction with their overall care.

The questionnaire was collected by the ward nursing staff on the day of discharge. The participants received a telephone follow-up within 7 days from a dedicated staff nurse to assess their symptom control and to address any concerns. Baseline demographic data like age, BMI, type of surgery and length of stay were collected from hospital records.

Data was entered prospectively into an excel database and was analysed using SPSS (version 6).

## Results

### Patients’ demographics

A total of 263 consecutive patients were involved in the study, with 144 (54%) patients completing and returning the questionnaires. Of the 144 patients, 50 (35%) patients had surgery for gynaecological cancers and 94 (65%) had surgery for benign gynaecological conditions (Table 2). Fifty-one percent (*n* = 74) of the patients had a laparoscopic hysterectomy for early endometrial cancer or benign pathology. Forty-nine percent (*n* = 70) had a laparotomy of which 46 patients had laparotomy for benign gynaecological conditions, and 24 patients had complex surgery for cytoreduction (Table 3).

**Table 2:** Diagnosis of patients

Diagnosis	Procedure	
	Laparoscopy (*N* = 74)	Open (*N* = 70)
Endometrial cancer	27	8
Ovarian cancer	1	7
Primary peritoneal cancer	0	3
Cervical cancer	4	0
Endometrial hyperplasia	6	0
Fibroid uterus	9	23
Benign ovarian cyst	13	16
Endometriosis	1	3
Risk reducing surgery	6	0
Menorrhagia	4	7
Pelvic pain	2	3
Severe dyskaryosis with incomplete colposcopy	1	0

Majority of the laparoscopic cases were performed for endometrial cancer whilst majority of the laparotomy cases were performed for fibroid uterus

**Table 3:** Types of procedure

Type of procedure	No. of cases (*n* = 144)
Laparoscopic hysterectomy for benign cases or early endometrial cancer	74
Open simple hysterectomy for benign cases (transverse and subumbilical incision)	46
Complex open surgery for staging/cytoreduction without bowel resection	23
Complex open surgery for staging/cytoreduction with bowel resection	1

The type and length of incision is known to impact patient’s recovery. Therefore, patients who had laparoscopic surgery with smaller incision have less pain and recovery quicker. Patients with a transverse and subumbilical incision would have less pain compared to patients with supraumbilical incision with complex open surgery

Patients who had laparoscopic surgery were significantly older compared to patients who had a laparotomy (54 versus 49; *p* = 0.004). The laparoscopic group had significantly short median length of stay when compared to the laparotomy group (1 versus 3 days; *p* = 0.001). There was no significant difference in BMI, ASA grade, indication for surgery, drain insertion and readmission rates. See Table 4 for patient demographics.

**Table 4:** Patient demographics (*N* = 144)

Variable	Procedure		Significance
	Laparoscopy (*N* = 74)	Open (*N* = 70)	
Age (median)	54 (31–91)	49 (21–83)	*p* = 0.004 *
Missing data	1	2	
BMI (mean)	27.9 (20.1–48.4)	27.9 (13.3–59.9)	*p* = 0.411
Missing data	17	15	
Length of stay (days) (median)	1 (0–5)	3 (1–13)	*p* = 0.001 *
ASA grade			
1 (a normal healthy patient)	24 (32.4%)	32 (45.7%)	*p* = 0.163
2 (a patient with mild systemic disease)	42 (56.8%)	31 (44.3%)	
3 (a patient with severe systemic disease)	6 (8.1%)	3 (4.3%)	
Missing data	2 (2.7%)	4 (5.7%)	
Indication			
Benign	42 (56.8%)	52 (74.3%)	*p* = 0.036
Cancer	32 (43.2%)	18 (25.7%)	
Drains			
Yes	12 (16.2%)	19 (27.15%)	*p* = 0.155
No	62 (83.8%)	51 (72.9%)	
Readmission			
Yes	7 (9.5%)	5 (7.1%)	*p* = 0.766
No	67 (90.5%)	65 (92.9%)	

*There was a significant difference in age and length of stay between the two groups, the laparoscopic group being of an older age group and having significant shorter length of stay

### Patient’s symptoms and their ability to achieve milestones set by ERAS

On the day of surgery, the laparoscopic group had significantly better pain control and were able to eat when they returned to the ward. In recovery, 58.1% (*n* = 43) of the laparoscopic group reported no pain compared with 27.2% (*n* = 19) of the open surgery group (*p* = 0.001). This difference in pain control continued when the patients were transferred to the ward with 37.8% (*n* = 28) of the laparoscopic group reported no pain compared with 7.1% (*n* = 5) in the laparotomy group (*p* < 0.0001). There was no difference in sickness control, tiredness, patient’s ability to perform the deep breathing exercises, sitting out of bed or in a chair and having a drink. The laparoscopic group were more confident in mobilising (20.3 versus 4.3%; *p* = 0.003) and in going home (29.7 versus 10.0%; *p* = 0.014) on the day of surgery compared with the laparotomy group (Tables 5, 6 and 7).

**Table 5:** Patients’ symptoms and patients’ satisfaction in recovery

Variable	Procedure		Significance
	Laparoscopy (*N* = 74)	Open (*N* = 70)	
Pain			
None	43 (58.1%)	19 (27.2%)	*p* = 0.001 *
Mild	16 (21.6%)	18 (25.7%)	
Moderate	11 (14.9%)	18 (25.7%)	
Severe	2 (2.7%)	11 (15.7%)	
Missing data	2 (2.7%)	4 (5.7%)	
Sickness			
None	50 (67.5%)	39 (55.7%)	*p* = 0.139
Mild	9 (12.2%)	14 (20.0%)	
Moderate	7 (9.5%)	12 (17.1%)	
Severe	6 (8.1%)	2 (2.9%)	
Missing data	2 (2.7%)	3 (4.3%)	
Warmth			
Cold	14 (18.9%)	10 (14.3%)	*p* = 0.768
Slightly cold	7 (9.5%)	9 (12.9%)	
Just right	38 (51.3%)	38 (54.2%)	
Warm	12 (16.2%)	8 (11.4%)	
Too warm	1 (1.4%)	2 (2.9%)	
Missing data	2 (2.7%)	3 (4.3%)	
Patient satisfaction in recovery			
Very dissatisfied	5 (6.7%)	4 (5.7%)	*p* = 0.28
Dissatisfied	0	1 (1.4%)	
Neutral	1 (1.4%)	4 (5.7%)	
Satisfied	15 (20.3%)	26 (37.1%)	
Very satisfied	50 (67.6%)	32 (45.8%)	
Missing data	3 (4.0%)	3 (4.3%)	

*Significantly more laparoscopic patients reported better pain control

**Table 6:** Patient’s ability to achieve milestones on the gynae ward on day 0

Variable	Procedure		Significance
	Laparoscopy (*N* = 74)	Open (*N* = 70)	
Have you done your breathing exercise?			
No	26 (35.1%)	23 (32.9%)	*p* = 0.726
Yes	46 (62.2%)	47 (67.1%)	
Missing data	2 (2.7%)	0	
Have you sat out of bed or sat out in a chair?			
No	42 (56.8%)	50 (71.4%)	*p* = 0.206
Yes	27 (36.5%)	19 (27.2%)	
Missing data	5 (6.7%)	1 (1.4%)	
Have you had a drink?			
No	6 (8.1%)	6 (8.6%)	*p* = 1.000
Yes	66 (89.2%)	63 (90.0%)	
Missing data	2 (2.7%)	1 (1.4%)	
Have you had something to eat?			
No	26 (35.1%)	38 (54.3%)	*p* = 0.041 *
Yes	45 (60.8%)	31 (44.3%)	
Missing data	3 (4.1%)	1 (1.4%)

**Table 7:** Patient’s symptoms and experience on the gynaecology ward on day 0

Variable	Procedure		Significance
	Laparoscopy (*N* = 74)	Open (*N* = 70)	
Pain control			
Not at all	28 (37.8%)	5 (7.1%)	*p* = 0.000 *
Mild	24 (32.4%)	22 (31.5%)	
Moderate	18 (24.4%)	32 (45.7%)	
Severe	0	7 (10.0%)	
Missing data	4 (5.4%)	4 (5.7%)	
Sickness control			
Not at all	45 (60.8%)	34 (48.5%)	*p* = 0.117
Occasionally	17 (23.0%)	23 (32.9%)	
Most of the time	3 (4.0%)	8 (11.4%)	
Severe	5 (6.8%)	2 (2.9%)	
Missing data	4 (5.4%)	3 (4.3%)	
Tiredness			
Not at all	4 (5.4%)	0	*p* = 0.100
Occasionally	24 (32.4%)	17 (24.3%)	
Most of the time	24 (32.4%)	32 (45.7%)	
Extremely	16 (21.6%)	16 (22.9%)	
Missing data	6 (8.2%)	5 (7.1%)	
Are you confident to mobilise?			
Not at all	27 (36.4%)	42 (60.0%)	*p* = 0.003 *
Occasionally	7 (9.5%)	13 (18.6%)	
Most of the time	10 (13.5%)	7 (10.0%)	
Completely	15 (20.3%)	3 (4.3%)	
Missing data	15 (20.3%)	5 (7.1%)	
Are you confident to go home?			
Not at all	35 (47.3%)	46 (65.7%)	*p* = 0.014 *
Occasionally	4 (5.4%)	7 (10.0%)	
Most of the time	5 (6.8%)	6 (8.6%)	
Completely	22 (29.7%)	7 (10.0%)	
Missing data	8 (10.8%)	4 (5.7%)	

*Significantly more patients in the laparoscopic group had early oral intake, better pain control and were more confident in mobilising and in going home

By day 1 post-operation, more patients in the open surgery group had moderate pain (54.3 versus 29.7%; *p* < 0.0001) and severe pain (12.9 versus 0%; *p* < 0.0001). Significantly more of the laparoscopic group reported no sickness compared with the open group (68.9 versus 44.2%; *p* = 0.003). However, despite the pain and sickness, more patients in the open surgery group performed the deep breathing exercises (87.1 versus 66.2%; *p* = 0.003) and they were just as able to sit out in the chair (82.9 versus 71.6%), have oral fluid (97.1 versus 89.2%) and food intake (90.0 versus 86.5%), walk 60 m and as frequently as the laparoscopic group. The laparotomy group remain less confident in mobilising independently (8.6 versus 39.2%; *p* < 0.0001) and going home (8.6 versus 52.7%; *p* < 0.0001) compared to the laparoscopic group (Tables 8 and 9).

**Table 8:** Patients’ ability to achieve milestones on the gynaecology ward on day 1

Variable	Procedure		Significance
	Laparoscopy (*N* = 74)	Open (*N* = 70)	
Have you done your breathing exercise?			
No	25 (33.8%)	9 (12.9%)	*p* = 0.003 *
Yes	49 (66.2%)	61 (87.1%)	
Missing data	0	0	
Have you sat out of bed or sat out in a chair?			
No	14 (18.9%)	11 (15.7%)	*p* = 0.511
Yes	53 (71.6%)	58 (82.9%)	
Missing data	7 (9.5%)	1 (1.4%)	
Have you had a drink?			
No	8 (10.8%)	2 (2.9%)	*p* = 0.98
Yes	66 (89.2%)	68 (97.1%)	
Missing data	0	0	
Have you had something to eat?			
No	10 (13.5%)	7 (10.0%)	*p* = 0.609
Yes	64 (86.5%)	63 (90.0%)	
Missing data	0	0	
Have you had a wash or shower?			
No	19 (25.7%)	12 (17.1%)	*p* = 0.230
Yes	55 (74.3%)	58 (82.9%)	
Missing data	0	0	
Are you able to put your own clothes on?			
No	6 (8.1%)	23 (32.9%)	*p* = 0.001 *
Yes	57 (77%)	43 (61.4%)	
Missing data	11 (14.9%)	4 (5.7%)	
Have you been seen by a physiotherapist today?			
No	6 (8.1%)	11 (15.7%)	*p* = 0.312
Yes	56 (75.7%)	58 (82.9%)	
Missing data	12 (16.2%)	1 (1.4%)	
How many times have you walked up to 60 m today?			
Nil	14 (18.9%)	23 (32.9%)	*p* = 0.123
Once	17 (22.9%)	20 (28.6%)	
Twice	14 (18.9%)	7 (10.0%)	
3 times	5 (6.8%)	11 (15.7%)	
4 times	7 (9.6%)	4 (5.7%)	
Missing data	17 (22.9%)	5 (7.1%)	

*Despite the pain and sickness, significantly more patients in the laparotomy group were able to perform the deep breathing exercises. Significantly more patients in the laparoscopic group were able to wear their own clothes

**Table 9:** Patient’s symptoms and experience on the gynae ward on day 1

Variable	Procedure		Significance
	Laparoscopy (*N* = 74)	Open (*N* = 70)	
Have you taken tablets for pain relief?			
No	4 (5.4%)	0	*p* = 0.058
Yes	61 (82.4%)	66 (94.3%)	
Missing data	9 (12.2%)	4 (5.7%)	
Pain control			
Not at all	13 (17.6%)	0	
Mild	31 (41.9%)	18 (25.7%)	*p* = 0.000 *
Moderate	22 (29.7%)	38 (54.3%)	
Severe	0	9 (12.9%)	
Missing data	8 (10.8%)	5 (7.1%)	
Sickness control			
Not at all	51 (68.9%)	31 (44.2%)	
Occasionally	10 (13.5%)	20 (28.6%)	*p* = 0.003 *
Most of the time	4 (5.4%)	13 (18.6%)	
Severe	3 (4.1%)	3 (4.3%)	
Missing data	6 (8.1%)	3 (4.3%)	
Tiredness			
Not at all	8 (10.8%)	2 (2.9%)	
Occasionally	33 (44.6%)	33 (47.1%)	*p* = 0.158
Most of the time	17 (23.0%)	25 (35.7%)	
Extremely	7 (9.4%)	6 (8.6%)	
Missing data	9 (12.2%)	4 (5.7%)	
Are you confident to mobilise?			
Not at all	3 (4.1%)	14 (20.0%)	
Occasionally	10 (13.5%)	27 (38.6%)	*p* = 0.000 *
Most of the time	20 (27.0%)	18 (25.7%)	
Completely	29 (39.2%)	6 (8.6%)	
Missing data	12 (16.2%)	5 (7.1%)	
Are you confident to go home?			
Not at all	11 (14.9%)	40 (57.1%)	
Occasionally	8 (10.8%)	13 (18.5%)	*p* = 0.000 *
Most of the time	7 (9.4%)	9 (12.9%)	
Completely	39 (52.7%)	6 (8.6%)	
Missing data	9 (12.2%)	2 (2.9%)	

*The above table shows there was better pain and nausea control in the laparoscopic group. In addition, the laparoscopic group was more able to dress independently and was more confident in mobilising and going home. However, the laparotomy group was more compliant with their breathing exercises

### Patients’ experience and satisfaction

Despite having different surgical procedures, both groups reported similar experience with the following aspects of their care. Both groups felt they were always treated with dignity and were well informed and involved in their treatment and care. They also received excellent care from nursing staff and doctors.

The patients’ differ in their experience of pain control and the overall satisfaction of care on the gynaecology ward. Significantly more patient in the laparoscopy group felt their pain was always well controlled (66.2% laparoscopic versus 48.6%; *p* < 0.0001). Finally, significantly more patients in the laparotomy group reported being satisfied to very satisfied with their overall care on the gynaecology ward (71.6% laparoscopic versus 81.4% laparotomy; *p* = 0.04) (Table 10).

**Table 10:** Patients’ overall experience and satisfaction

Variable	Procedure		Significance
	Laparoscopy (*N* = 74)	Open (*N* = 70)	
Were you given a leaflet on enhanced recovery?			
No	7 (9.5%)	8 (11.4%)	*p* = 1.000
Yes	43 (58.1%)	49 (70.0%)	
Missing data	24 (32.4%)	13 (18.6%)	
Were you treated with dignity?			
Never	0	0	
Sometimes	0	2 (2.9%)	*p* = 0.496
Always	55 (74.3%)	56 (80.0%)	
Missing data	19 (25.7%)	12 (17.1%)	
Did you feel well informed about your treatment?			
Never	0	0	*p* = 0.054
Sometimes	2 (2.7%)	9 (12.9%)	
Always	53 (71.6%)	49 (70.0%)	
Missing data	19 (25.7%)	12 (17.1%)	
Did you feel your pain was well controlled?			
Never	0	0	
Sometimes	5 (6.8%)	22 (31.4%)	*p* = 0.000 *
Always	49 (66.2%)	34 (48.6%)	
Missing data	20 (27.0%)	14 (20.0%)	
Did you feel you were involved in decisions about your discharge from hospital?			
Never	0	2 (2.9%)	*p* = 0.154
Sometimes	3 (4.1%)	6 (8.6%)	
Always	51 (68.9%)	42 (60.0%)	
Missing data	20 (27.0%)	20 (28.5%)	
How would you rate the friendliness, approachability and respect of the nursing staff?			
Poor	0	0	*p* = 0.709
Adequate	1 (1.4%)	2 (2.9%)	
Good	11 (14.8%)	8 (11.4%)	
Excellent	49 (66.2%)	43 (61.4%)	
Missing data	13 (17.6%)	17 (24.3%)	
How would you rate the friendliness, approachability and respect of the doctors?			
Poor	0	0	*p* = 0.431
Adequate	1 (1.4%)	0	
Good	11 (14.8%)	6 (8.6%)	
Excellent	49 (66.2%)	47 (67.1%)	
Missing data	13 (17.6%)	17 (24.3%)	
How would you rate your overall stay on the gynaecology ward?			
Very dissatisfied	0	0	*p* = 0.04 *
Dissatisfied	1 (1.4%)	0	
Satisfied	9 (12.2%)	20 (28.6%)	
Very satisfied	44 (59.4%)	37 (52.8%)	
Missing data	20 (27.0%)	13 (18.6%)	

*Apart from pain control and patients’ satisfaction with their stay on the gynaecology ward, there was no significant difference between the rest of patients’ experience and satisfaction with their care

## Discussion

This study has shown the laparotomy group had poorer pain and sickness control and were less confident in mobilising and going home. Despite their symptoms, the laparotomy group were better at performing the deep breathing exercises. Both groups were equally able to achieve the ERAS milestones in drinking, mobilising, and sitting out in a chair. Apart from pain control and patients’ satisfaction with their stay on the gynaecology ward, there was no significant difference between the rest of patients’ experience and satisfaction with their care.

Patients’ confidence in mobilising and going home improved in both groups from day 0 to day 1. However, the laparoscopic group remain significantly more confident in mobilising and in going home. This may be a consequence of the laparoscopic group experiencing less pain, thus enabling them to return to normal daily function such as ability to mobilise and put on their own clothes which increases their confidence in mobilising and going home.

The enhanced recovery after surgery (ERAS) programme was introduced by the department of health to improve the quality of care through improving patients’ clinical outcomes and experience. This programme has shown to be cost-effective with high patient satisfaction and shorted length of stay (DH, N.I., NCAT, NHS institute 2011; Blazeby et al. 2010). There is limited data in literature evaluating patients’ experience and functional recovery of activities of daily living following surgery. Functional recovery after ERAS comparing laparoscopy and laparotomy approach has been evaluated in two studies in patients undergoing colonic resection, but there are no studies in patient’s undergoing gynaecological surgery (Basse et al. 2005; King et al. 2006). Basse et al. 2005 evaluated functional recovery parameters such as hours of mobilisation per day, computerised monitoring of motor activity, pain, fatigue, sleep quality, pulmonary function, cardiovascular response to treadmill exercise and return to normal gastrointestinal function between laparoscopy and laparotomy group in a randomised study involving 60 patients. Interestingly, they showed that laparoscopic group experienced higher pain on days 0 and 1 compared to the laparotomy group but there was no difference between the groups after day 2. Nausea and vomiting was similar in both groups. There was early normalisation of gastrointestinal function within 2 days in both groups. Both groups were out of bed for median duration of 10 h on day 1. Computerised monitoring of motor activity confirmed a high degree of mobilisation in both groups. The authors concluded that functional recovery after colonic resection is rapid with ERAS without difference between open and laparoscopic resection. King et al. 2006 assessed functional recovery based on mobility, balance, gait, lower extremity strength and endurance to produce a performance score. In this randomised study of 62 patients, assessment of functional recovery was conducted by more than 80% of patients at intervals up to 12 weeks postoperative. In both groups, the performance score deteriorated after surgery and had not returned to pre-operative value by 3 months. Moreover, the laparotomy group remained significantly worse. Therefore, King et al. concluded that the laparotomy group had poorer functional recovery. Our findings on pain, nausea and sickness control differed from Basse et al.’s study. In our study, the laparoscopic group had better pain control in recovery which continued to day 1. It is well known that laparoscopic surgery is associated with less pain and ileus due to smaller incisions leading to quicker recovery compared to laparotomy (Wodlin and Nilsson 2013; O'Dwyer et al, 1992). Therefore, significantly more patients in the laparoscopic group had something to eat on day 0. Both groups had good sickness control in recovery and on day 0. However, on day 1, the laparotomy group had poorer sickness control. This may be as a result of the anaesthetic wearing off producing more pain and sickness. By day 1 the laparotomy group were just as able to have a drink and something to eat, sit out in a chair, have a shower, and mobilise. The laparoscopic group were better at putting on their clothes and were more confident in mobilising and going home. Our study found both groups were able to perform majority of the milestones despite significant differences in their pain, nausea and sickness control and their confidence in mobilising and going home. This may be a reflection of how patient education can help patients understand and cope with their symptoms. This in turn empowers patients to play a proactive role in their recovery thus enabling them to achieve the ERAS milestones.

Our paper was consistent with King et al.’s finding where the short- and medium-term outcomes were better following laparoscopic surgery. However, King et al. had a longer follow-up period for up to 12 weeks.

Interestingly, the laparotomy group were better at performing the deep breathing exercises in spite of having significantly more pain. This may be a result of effective patient education at pre-operative clinic. During the study, we identified that only the laparotomy group was seen by a physiotherapist at the pre-operative clinic. This was due to limited funding. They were shown how to perform the deep breathing exercises effectively and how to get out of bed safely. The laparoscopic group were only given a leaflet on how to perform deep breathing exercises. A qualitative study of 14 patients by Archer et al. 2014 found availability of information and physio at pre-op plays an important part in order to help patients understand why they were asked to comply with the programme and to help set expectations. The authors concluded that pre-operative education can help patients with self-motivation and empowers them to participate actively in their recovery.

Blazeby et al. 2010 in their qualitative study comparing 14 patients undergoing laparoscopic surgery and laparotomy for colorectal cancer showed increased satisfaction for early discharge and rapid return to baseline function. They have also highlighted that improved access to information and specialist advice at discharge improves patients’ satisfaction. There has been only one study evaluating patients’ satisfaction with ERAS in gynaecological oncology (Philp et al. 2015). Philp et al. in their study of 207 patients undergoing laparotomy for gynaecological cancer in the ERAS programme reported high patient satisfaction with doctors and nurses caring for them post operatively. In addition, providing patient information about discharge also played important part in quicker recovery and satisfaction. We have shown similar findings of high level of satisfaction in the care provided by doctors and nurses with good experience of their stay on the ward, and patients’ satisfaction remains high in both laparoscopic and laparotomy group even in the early stages of recovery.

The strength of this study is that this is the first prospective study to compare functional recovery based on ability to achieve milestones and patients’ satisfaction in laparoscopic versus open gynaecological surgery. In spite of the questionnaires being given out on day of admission and collected before discharge, the response rate was only 54%. There was no obvious reason for the low response rate given that this was a questionnaire completed whilst in hospital and returned on discharge. We limited the evaluation of the study to day 1 post operation as most of the laparoscopic cases were discharged the day after surgery, and we felt that making a comparison between two groups beyond day 1 was not comparable.

## Conclusion

We have shown that within the ERAS programme for patients undergoing gynaecological surgery, the two groups were in general equally able to achieve the majority of the milestones despite difference in symptoms such as pain, nausea and confidence in mobilising and going home. Pain is likely to affect more complex activity like putting on one’s own clothes and patients’ confidence in mobilising and going home. Pre-operative education can empower patients to engage in their recovery, and there is a high level of patient satisfaction in both groups.

Future studies should focus more closely on why some patients chose not to participate. In addition, a future longitudinal study comparing patients’ post-operative recovery focusing on ability to perform activities of daily living at 1, 2 and 4 weeks may give us a better understanding regarding the short-term recovery of these patients.

## Abbreviations

BMI: Body mass index
ERAS: Enhanced recovery of surgery
ERP: Enhanced recovery programme
NSAIDS: Non-steroidal anti-inflammatory drug
TIVA: Total intravenous anaesthesia
